# Thoughts of Death Modulate Psychophysical and Cortical Responses to Threatening Stimuli

**DOI:** 10.1371/journal.pone.0112324

**Published:** 2014-11-11

**Authors:** Elia Valentini, Katharina Koch, Salvatore Maria Aglioti

**Affiliations:** 1 Sapienza Università di Roma, Dipartimento di Psicologia, Roma, Italy; 2 Fondazione Santa Lucia, Istituto di Ricovero e Cura a Carattere Scientifico, Roma, Italy; University of Waterloo, Canada

## Abstract

Existential social psychology studies show that awareness of one's eventual death profoundly influences human cognition and behaviour by inducing defensive reactions against end-of-life related anxiety. Much less is known about the impact of reminders of mortality on brain activity. Therefore we explored whether reminders of mortality influence subjective ratings of intensity and threat of auditory and painful thermal stimuli and the associated electroencephalographic activity. Moreover, we explored whether personality and demographics modulate psychophysical and neural changes related to mortality salience (MS). Following MS induction, a specific increase in ratings of intensity and threat was found for both nociceptive and auditory stimuli. While MS did not have any specific effect on nociceptive and auditory evoked potentials, larger amplitude of theta oscillatory activity related to thermal nociceptive activity was found after thoughts of death were induced. MS thus exerted a top-down modulation on theta electroencephalographic oscillatory amplitude, specifically for brain activity triggered by painful thermal stimuli. This effect was higher in participants reporting higher threat perception, suggesting that inducing a death-related mind-set may have an influence on body-defence related somatosensory representations.

## Introduction

*"[…] Death, the most dreaded of evils, is therefore of no concern to us; for while we exist death is not present, and when death is present we no longer exist. Epicurus (Letter to Menoeceus, 43–44).*

Awareness of unavoidable death has a powerful impact on cognition and human behaviour [1]. The terror management theory (TMT) has shown that pondering on one's own mortality promotes stereotypical thinking as well as a defensive attitude towards one's own values and beliefs [2]. An important asset of the TMT is the hypothesis that cultural and personality factors may act as mediators of the anxiogenic effects caused by the awareness of death. In particular, the hypothesis that increased self-esteem makes an individual less prone to anxiety or thoughts about death has received much experimental support (e.g. [3]–[6]). Other authors have shown how priming thoughts about one's death induces negative emotions (e.g. anxiety), provokes avoidance of self-focused states [7], and leads individuals high in neuroticism to avoid physical sensations, including pleasurable ones [8].

Although there is general agreement that thoughts of death significantly affect cognition and human behaviour, only a few studies have investigated how thoughts of death influence cortical representation of sensory information. Noteworthy here are studies that investigated the effect of death-content accessibility on bold signal or event-related potentials (ERPs) amplitudes [9]–[11] and on its interaction with neural processes linked to social-affective categorization of facial expressions [12], as well as with observation of others' pain [13]. More specifically, using fMRI, Quirin et al. [10] reported that accessibility to thoughts of death induced higher activation of structures usually associated with emotion regulation, such as the amygdala and the anterior cingulate cortex (ACC). More recently, Klackl et al. [11] reported larger late positive potential amplitudes associated with death-related words, a finding that may be interpreted as indexing preferential mortality salience effects on emotion regulation. Nevertheless, these results are in contrast with evidence of decreased ACC and insular activity for death-related words in the context of a linguistic Stroop task [9]. Surprisingly, although a relationship between mortality salience effects and implicit anxiogenic mechanisms has been acknowledged in previous studies, there is currently no evidence linking the effects of mortality salience to representation of threatening sensory information within the central nervous system.

Here, we sought to determine whether thoughts of death can influence perceptual ratings and cortical representations associated to threatening sensory stimuli. Combining a paired stimulation design with electroencephalography (EEG), we explored the effects of mortality salience on ERPs and oscillatory theta activity elicited by pairs (S1–S2) of laser thermal painful stimuli, before and after the induction of a cognitive mind-set (i.e., a mental disposition). Pairs of auditory stimuli, matched in subjective intensity with the painful radiant thermal stimuli, were used to explore the effects on perception and neural representation of non-painful stimuli. Paired stimulation was conceived as a minimalist approach to induce a repetition suppression effect (e.g. [14]). Indeed, most of studies investigating short-term habituation of EEG responses evoked by radiant heat stimulation reported a dramatic reduction of response magnitude to repeated identical stimuli already at the level of the second stimulus, with no further decrement in response to the following stimuli (e.g. [15], [16]). The second stimulus of each pair was therefore designed as a test stimulus and conceived as a minimal measure of basic sensitization/habituation processes.

We hypothesized that mortality salience interferes with phasic cortical responses to repeated threatening sensory stimuli by exerting a top-down allocation of attentional resources regardless of sensory stimulation salience that leads to stimulus detection and attentional orientation processes [17], and thus impairing the reduction of response amplitude observed to repeated stimulation at short fixed inter-stimulus interval (e.g. [18], [19]).

Overall, the present design enabled us to isolate the effects of mortality salience from i) the sole salience or novelty of the sensory stimulation, ii) the variability of the neural responses prior to the mind-set induction, and thus iii) disclose cognitive/emotional top-down modulations of cortical representation of threat contingent upon accessibility to death thoughts.

## Methods

### Ethics statement

Participants gave written informed consent and were debriefed at the end of the experiment. All experimental procedures were approved by the Fondazione Santa Lucia local ethics committee and were in accordance with the standards of the Declaration of Helsinki.

### Participants

Twenty right-handed healthy participants (12 females) aged between 21 and 33 (mean ± SD, 24.5±4.4) participated in the study. All had normal or corrected-to-normal vision and were naïve as to the purpose of the experiment. None of the participants had a history of neurological or psychiatric illnesses or conditions that could potentially interfere with pain sensitivity (e.g. drug intake or skin diseases).

### Personality measures

Preliminary screening and selection of volunteers was conducted using self-report measures of personality traits that could potentially interfere with the effect of the applied mind-set induction on perception and cortical arousal. The Beck Depression Inventory (BDI) [20] and the State-Trait Anxiety Inventory (STAI) [21] were administered to obtain an index of individual psychopathological symptoms of depression and anxiety, respectively. Participants who scored higher than 17 on the BDI and higher or lower than two standard deviations (SD) on the STAI were not allowed to enter the study [22]. These cut-off scores determined the preliminary exclusion of two participants.

### Nociceptive and auditory stimulation

The nociceptive heat stimuli were pulses generated by an infrared neodymium yttrium aluminium perovskite (Nd:YAP) laser with a wavelength of 1.34 µm (Electronical Engineering, Florence, Italy). Duration of the laser pulses was 5 ms. These pulses selectively and directly activate the Aδ and C-fiber nociceptive terminals located in the superficial layers of the skin [23]_ENREF_4. The laser beam was transmitted via an optic fiber and its diameter was set at approximately 7 mm (≈38 mm^2^) by focusing lenses. Laser pulses were delivered on a square area (5×5 cm) defined on the left hand dorsum prior to the beginning of the experimental session. He-Ne laser indicated the area to be stimulated. To prevent increases in baseline skin temperature and fatigue or sensitization of the nociceptors, the position of the laser beam was changed after each pulse. An infrared thermometer (precision ±0.3°C) was used to measure the temperature of the stimulated skin area before and during the experiment (group-average intensity of 34.2±0.7°C). Temperature fluctuations never exceeded 0.8 SD°C within participants.

During a familiarization and calibration procedure on the quality of the sensation associated with radiant heat stimuli, participants were instructed to define the intensity of the sensation using both a numerical rating scale (NRS) and a visual-analogue scale (VAS). For both of these methods, intensity was defined as how strong the sensation was. Participants were instructed to verbally rate intensity of painful stimuli according to the NRS from not intense or barely intense (0–10) to low intense (21–40), moderately intense (41–60), highly intense (61–80) and extremely intense (81–100). Participants were allowed to give decimal ratings over the entire numerical scale. The energy of the stimulus was adjusted using a staircase procedure. The procedure required one increase (increasing) series and one decrease (decreasing) series in steps of 0.5 Joules (J), followed by an increase (increasing) series in steps of 0.25 J until the target intensity of the nociceptive-related sensation was reported (i.e. pricking/burning sensation; [24]). Lastly, energies within 0.5 J below and above the energy eliciting the pricking/burning sensation were delivered to test the reliability of the intensity ratings. Eventually, all the calibrated stimuli were defined as painful by the participants and perceived as threatening. As our objective was to establish a perceptual similarity between laser heat- and auditory-related percepts, once the target intensity was found and the corresponding laser energy calibrated (group-average intensity of 4.5±0.4 J), participants were required to self-adjust the intensity of the auditory stimulation to match the intensity of the nociceptive stimulus using the same criteria as the NRS for the nociceptive stimuli (see [19], [25] for a detailed description). This procedure was applied to create a threatening experience similar to the one induced by somatosensory nociceptive stimuli, by asking the participants to focus on the most simple aspect of somatosensory nociceptive sensation: its magnitude. By matching the two types of sensory stimuli according to their magnitude we obtained a match of the salience of sensory stimulation and reduced the complexity of a matching procedure based on cognitive/affective aspects of the stimuli (e.g. unpleasantness), while obtaining a comparable level of threat for auditory stimuli during the experiment. Auditory stimuli were short tones of 800 Hz frequency (50 ms; 5 ms as the rising and falling time of the tone) emitted by a loudspeaker placed in front of the participants' left hand (≈50 cm from the participant and ≈50 cm from the midline). Once auditory intensity was calibrated (group-average intensity of 81.8±3.6 dB; measured at the subject's left ear), participants underwent a brief learning procedure during which the NRS anchors were transferred onto the experimental VAS. If a significant discrepancy was noticed between NRS ratings during calibration and VAS judgments during learning, the calibration procedure was repeated.

During this procedure the participants received paired stimuli (at a fixed interval of 1 s) to accurately match the two modalities according to the requirements of the experimental design.

### EEG recording

EEG recordings were obtained from sixty tin electrodes (Electro-cap International - ECI) placed according to the positions of the 10–20 International System. Three surface electrodes were positioned for the vertical, horizontal electro-oculography (EOG) recording below the right eye and at the right and left ocular canthi and one electrode at the left mastoid for electromyography recording (EMG). The reference was on the nose and the ground at AFz. Electrode impedance was kept below 5 kΩ. The EEG signal was amplified and digitized at 1000 Hz.

### Design and experimental procedure

**Figure 1:**
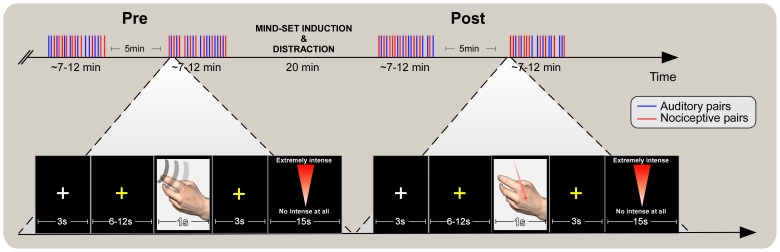
Experimental design. EEG activity and subjective ratings of intensity and threat of sensory stimulation were collected in two separate experimental sessions during which participants underwent a 'Mortality Salience' (MS) or an 'Exam Salience' (ES) mind-set induction (top panel, central). The order of MS and ES was counter-balanced across participants. ERPs elicited by either nociceptive somatosensory stimuli delivered to the hand dorsum (top panel, red) or by auditory stimuli delivered in the same area (top panel, blue) were recorded in four blocks. The first two blocks were free from cognitive manipulation (condition 'Pre', top left) whereas the following two blocks (condition 'Post', top right) were preceded by the mind-set induction (5 min) and a distraction period (20 min) during which participants completed the Positive and Negative Affect Schedule and the State Anxiety Inventory. In each block, 20 pairs of stimuli (S1–S2, a pair), 10 per each sensory modality, were delivered in a pseudorandom order. The stimuli composing a pair were separated by 1 s inter-stimulus interval. Each pair established a single trial which started with a fixation cross on the screen (3 sec), followed by a yellow fixation cross (6–13 sec) in which the pair was jittered (bottom panel). Three seconds after receiving each pair of stimuli, participants were required to rate (on a 101-point electronic visual-analogue scale) the intensity and threat of each stimulus in the pair (thus providing two ratings for S1 and two for S2 within a 15 sec time window). This procedure allowed determining whether any modulation was exerted by the mind-set induction on the cortical responses and perception associated with nociceptive and auditory stimuli.

Participants underwent two separate experimental sessions (on two different days, same time of day). In both sessions, participants were submitted to four recording blocks (Fig. 1, top panel). The first two blocks had no cognitive manipulation (condition *pre*) and served as a baseline condition to compare with the modulatory effects of the following cognitive manipulation (condition *post*). After the first two blocks, participants were randomly assigned to one of two mind-set conditions (cf. [26], [27]). The order in which the mind-sets were administered in the two experimental sessions was counterbalanced across participants (Fig. 1, top panel, centre). Following the typical TMT paradigm, participants were asked to write down their thoughts in a short questionnaire consisting of two open questions that focused either on the possibility of their own death ('Mortality Salience'-*MS*) or the contingency of having failed an important exam ('Exam Salience'-*ES*). The *ES* mind-set induction was meant to trigger a negative valence state similar to that induced by the *MS* condition; thus, it controlled for the specific effects of mortality salience on human behaviour [27], [28]. Importantly, *ES* was selected as the control condition after a preliminary pilot survey in which several different mind-sets used in the experimental TMT literature were compared along different dimensions in a sample of 100 respondents. *ES* was judged as the condition most similar to *MS* (thus, not significantly different from it) across several parameters, e.g. arousal, valence, threat, puzzlement (see Material S1). Participants had 5 min to answer the questions, after which they were exposed to a distraction period. This was based on the notion that to observe the implicit effects associated with mortality salience the individual should be distracted from the salience of this mental content [29]. The distraction period lasted 15 min during which participants completed the Positive and Negative Affect Schedule (PANAS, [30]) and the State Anxiety Inventory (STAI-Y, [31]), and were asked to play with a brain-shaped Rubik's cube before undergoing the EEG again (20 minutes in total). Administration of the questionnaires was repeated immediately after the last two blocks to check for carry-over effects caused by the mind-set induction on participants' self-reported state mood and anxiety.

Participants were comfortably seated in a temperature-controlled room (25 C°) with their hands resting on a table, ≈40 cm from the body midline. A wooden frame blocked the sight of their left arm and the laser device. Participants were asked to relax and fixate the center of the computer screen placed in front of them. The background of the computer screen was black throughout the experiment. Each block lasted between 7 and 12 min and there was a 5 min pause between blocks 1 and 2 and blocks 3 and 4 (Fig. 1, top panel). In each block, 20 pairs of stimuli (S1–S2, a pair), 10 per each sensory modality, were delivered in a pseudo-random fashion (no more than three consecutive pairs belonging to the same modality) or near the left hand dorsum at a constant inter-stimulus interval (ISI) of 1 s. Thus, each participant was subjected to 80 trials (40 pairs and 80 single stimuli per modality) in each experimental session. Between each laser pulse of a nociceptive pair, the laser beam was manually displaced by at least 1 cm along a proximal-distal line on the hand dorsum [15]. The direction of this displacement was balanced in each block (10 pairs in the proximal direction and 10 pairs in the distal direction). A proximal-distal spatial displacement was used to minimize the role of variations in thickness and innervations of the irradiated skin [32] in affecting the strength of the nociceptive input.

Each pair formed a single experimental trial (Fig. 1, bottom panel) in which S1 was considered the conditioning stimulus and S2, the test stimulus. The timing of each trial was as follows; a white fixation cross on the computer screen (3 s) was followed by a yellow fixation cross that alerted the participants to relax all their muscles and avoid eye movements before the impending stimulation (6–12 s). Nociceptive and auditory stimuli composing the pair were delivered at 1 s inter-stimulus intervals and jittered during this time window. After the delivery of each pair of stimuli a yellow fixation cross appeared on the screen (3 s) to signal participants to wait to report their sensations. Participants were asked to rate both intensity and threat for each stimulus in the pair (i.e., provide two ratings for S1 and two for S2) using the right hand to move a mouse and position a pointer on a 101 point electronic visual-analogue scale (VAS) on the screen, within 15 s from its appearance. At the bottom of this scale, zero was represented by the label “not intense at all” for the intensity assessment and “not threatening at all” for the threat assessment. At the top of this scale, 100 was represented by the label “extremely intense” or “extremely threatening”. Intensity and threat ratings were asked in a pseudo-random order (repeated no more than three times within each block). Threat was defined during the brief learning procedure and was meant to distinguish the sensory-discriminative dimension associated with the magnitude of the sensation from a cognitive-affective dimension related to interpretation of its homeostatic meaning. Threat ratings were measuring participants' interpretations of the stimuli as indicating imminent danger, warning of an incoming unpleasant state. According to the trial timeline, the inter-trial interval thus ranged between 24 and 30 s. During *pre* and *post* blocks, the group's average skin temperature was 34.2±0.7°C and 34.3±0.9°C, respectively.

### Data analysis

#### State mood and anxiety

Scores obtained on the PANAS and STAI scales were analyzed using the Wilcoxon matched pairs test to compare scores obtained immediately after *MS* and *ES* inductions as well as at the end of the experiment. The level of significance was set at *P*<0.05.

#### Psychophysics

The calibration procedure was aimed at improving participants' ability to detail their sensations and concurrently counteract the ordinal nature of the VAS scale by increasing within and between subjects reliability [33]. This approach allowed for the normal distribution of intensity ratings in all conditions across the two different sensory modalities. Indeed, participants could be clustered in three different ranges of perceived intensity: a lower bound, corresponding to a sensation of low intensity (21–40; mean and SD  = 34.7±4.1; n = 4), a middle range corresponding to moderate intensity (41–60; 51.7±6.4; n = 10) and an upper bound corresponding to high intensity (61–80; 71.1±7.5; n = 6).

The factor Time (two levels: *pre* and *post*) was split in order to feed an analysis of covariance (ANCOVA) with a continuous predictor *pre* and a categorical predictor Mind-set (two levels: *MS* and *ES*) of the response pattern observed in the *post* mind-set induction measures (i.e., the dependent variables). ANCOVA was carried out separately on ratings obtained in each sensory modality (auditory and nociceptive). Analysis of covariance is the most powerful statistical approach for experiments in which subjects are assigned randomly to treatment groups, regardless of whether there is a bias due to the initial measurement, because it allows reducing within group error variance (i.e. it strongly reduces between-subject variability from the treatment comparison) [34]–[37]. Finally, we computed *post-pre* change scores for both *MS* and *ES* conditions; they were compared using t-tests for paired dependent samples. The level of significance was set at *P*<0.05. Partial eta squared (pη^2^) as measures of effect size of significant main effects and interactions are reported.

#### EEG preprocessing

EEG data were preprocessed with Vision Analyzer software (Brain Products, v. 1.05). They were first downsampled to 250 Hz, transformed to the average reference [38], DC detrended and band-pass filtered from 0.5 to 30 Hz. Data were then segmented into epochs using a time window ranging from 1 s before the first stimulus (S1) to 1 s after the second stimulus (S2) of each pair (total epoch duration: 3 s). Epoched data were further processed using EEGLAB (v. 12.x; [39]) and Letswave 5 (http://nocions.webnode.com/). EOG and EMG artifacts were subtracted using independent component analysis (ICA; [40]).

#### EEG Analysis in the time domain

Epochs belonging to the same experimental condition (*ES pre, ES post, MS pre, MS post*) were averaged and time-locked to the onset of the first stimulus of each pair. This procedure yielded eight average waveforms, one for each experimental condition and sensory modality (nociceptive and auditory respectively). For each individual average waveform, the relative peak amplitude of the late nociceptive and auditory evoked potentials (NEPs and AEPs respectively) elicited by S2 was extracted (mean of 10 ms around the peak). For NEPs N1, the mean of the activity in the range of the observed topography was extracted (130–180 ms). The NEP N1 wave was measured at the temporal and central electrodes contralateral to the stimulated side (T8 and C4), referenced to Fz (see [41], [42]). It was defined as the negative deflection preceding the N2 wave, which appears as a positive deflection in this montage. The N2 and P2 waves were measured at the vertex (Cz) referenced to the common average. The N2 wave was defined as the most negative deflection after stimulus onset. The P2 wave was defined as the most positive deflection after stimulus onset. For AEPs, N1 and P2 waves were measured at the vertex (Cz) referenced to the common average. The N1 wave was defined as the most negative deflection after stimulus onset. The P2 wave was defined as the most positive deflection after stimulus onset.

ANCOVA was carried out separately on the extracted S2 NEP and AEP amplitudes. Then, whole-waveform t-tests (i.e. the entire EEG signal in a given epoch) were performed to assess point-by-point amplitude differences within each mind-set (*pre* vs. *post*) and the differences between the two mind-sets during *pre* and *post* induction (*MS* vs. *ES*) on the S2 evoked activity. The threshold for statistical significance was set at *P*<0.05. Furthermore, differences in amplitude intervals were considered as significant only when they lasted at least 10 ms, a temporal cluster used to account for multiple comparisons. The maximal *t* value (signed) in each relevant time interval is reported. These analyses allowed testing the relevant differences within (*pre*-*post* increase vs. decrease in amplitude) and between (increase vs. decrease in amplitude within *MS* or *ES*) conditions. The finding of a difference in ERPs amplitude between *pre* and *post*, regardless of the mind-set induction, points to an unspecific mind-set effect on repetition suppression. Conversely, the finding of a difference during *post*-induction trials only when there was no difference during *pre*-induction trials suggests a specific effect of mind-set on repetition suppression.

#### EEG Analysis in the time-frequency domain

Time-frequency representations (TFRs) were computed using a Morlet wavelet in which the initial spread of the Gaussian envelope was set at 0.15 and the central frequency of the wavelet at 3 Hz. The transform expressed the oscillation amplitude as a function of time and frequency, regardless of its phase [43]. Averaging these estimates across trials discloses both phase-locked and non-phase-locked modulations of signal amplitude. Across-trial averaging of these time–frequency representations produced a spectrogram of the average EEG oscillation amplitude as a function of time and frequency. For each estimated frequency, results were displayed as an event-related percentage (ER%) increase or decrease in oscillation amplitude relative to a pre-stimulus reference interval (−0.6 to −0.2 s before the onset of S1), according to the following formula: ER*_t,f_* %  =  [A*_t,f_* - R*_f_*]/R*_f_*, where A*_t,f_* is the signal amplitude at a given time *t* and at a given frequency *f*, and R*_f_* is the signal amplitude averaged within the reference interval [44]. Recent studies confirmed that the theta and gamma frequency bands (e.g. [45], [46]) reflect aspecific and specific information related to pain perception. Here we focused on the theta frequency range (3–8 Hz). Thus, one time-frequency region of interest (ROIs) was defined in the spectrograms obtained at Cz, where the main spectral events maximally express their magnitude. The time-frequency limits of the time-frequency ROI (3–8 Hz and 100–500 ms) were defined according to previous studies (e.g. [47], [48]). Within the time-frequency ROI, ER% amplitudes were extracted by computing the mean of the 10% pixels displaying the highest activity in the given time-frequency range. This “top 10%” summary measure reflects the higher ER% values within each window of interest to reduce the noise introduced by including near-to-zero values. This approach, which was successfully used to analyze both EEG [15] and fMRI data [49], [50], proved suitable to disclose condition-specific effects [19], [51]–[53]. For point-by-point t-tests, the same data analysis approach implemented in the time domain was used in the time-frequency domain; the only exception was the temporal cluster chosen for significance: amplitude intervals were considered as significant only when they lasted more than 20 ms.

#### Additional analyses

Gender, age, measures of mood and anxiety as well as ratings of intensity and threat were used as categorical or continuous covariates in separate ANCOVAs in which, together with *pre* mind-set activity, their contribution to the significant differences observed between *MS* and *ES* summary measures was tested.

In addition, observed differences were further assessed by testing the moderating effect of an amplitude response profile (ARP) in each individual. This was at variance with the use of the sole regressor pre for S2 activity, as the ARP was calculated as the difference of S1–S2 activity in *pre* blocks. Specifically, participants were split into low and high amplitude suppressors (*lows*, *highs*) according to the median value of the mean activity recorded in the *pre* mind-set induction blocks. *Lows* and *highs*, classified according to a median split procedure, were considered as two levels of a categorical predictor (Suppressors), which entered ANCOVA with the continuous regressor *pre*, the categorical predictor Mind-set (two levels: *MS* and *ES*) and S2 peak amplitudes as the dependent variable. All the additional analyses were computed only on the neural activities affected by Mindset according to the main ANCOVA analyses.

## Results

### State mood and anxiety

*Pre* and *post* state mood and anxiety score distributions were not significantly different between *MS* and *ES* conditions immediately after mind-set induction (PANAS positive: *Z* = 0.59, *P* = 0.55; PANAS negative: *Z* = 1.54, *P* = 0.12; State anxiety: *Z* = 1.54, *P* = 0.12) or at the end of the experimental session (PANAS positive: *Z* = 0.67, *P* = 0.50; PANAS negative: *Z* = 0.12, *P* = 0.91; State anxiety: *Z* = 0.35, *P* = 0.72).

Thus, there was no difference in aware feelings of mood or anxiety between the two different mind-sets, which suggests a similar activation of proximal defenses [54].

### Nociceptive intensity and threat

**Figure 2:**
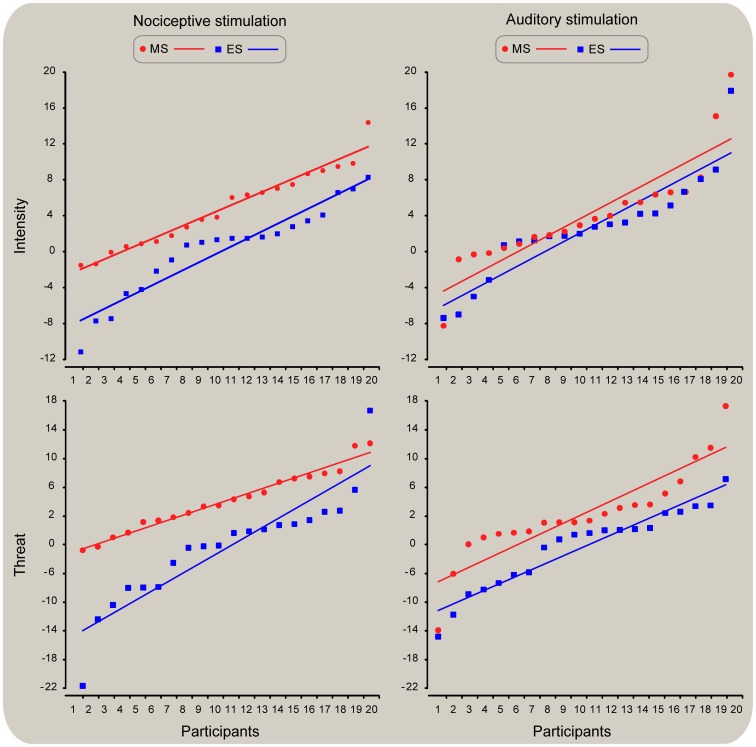
Scatterplots of mindset-induced changes (Post-Pre) in rating intensity and threat of S2 for both nociceptive and auditory stimuli. The x axis shows each participants' ratings as a function of increased range of intensity and threat. The corresponding average ratings of intensity and threat are displayed on the y axis. Negative and positive values indicate lower and higher ratings following mind-set induction. Individual data were fitted by a linear function. An increase of both intensity and threat was observed during the MS condition, especially in the nociceptive modality (left panel).

All the stimuli were perceived as painful by participants. The ANCOVA on intensity and threat ratings at S2 revealed that the covariate *pre* was significant for the analysis of intensity and threat (*F*(1, 37) = 410.33, *P*<0.001; pη^2^ = 0.92 and *F*(1, 37) = 638.78, *P*<0.01; pη^2^ = 0.94, respectively). When adjusting for the effect of the ratings obtained in the *pre* blocks, a significant main effect of Mind-set was observed in intensity and threat ratings in the *post* blocks (*F*(1, 37) = 9.92, *P*<0.01; pη^2^ = 0.21 and *F*(1, 37) = 11.88, *P*<0.01; pη^2^ = 0.24, respectively). This effect was accounted for by higher ratings of intensity and threat in *MS* trials than in *ES* trials, as confirmed by the paired t-tests performed on *post - pre* change scores (*t*(19) = 3.93, *P*<0.01, and *t*(19) = 4.37, *P*<0.001 respectively) (Fig. 2, left panel). Importantly, when controlling for the effect of the three ranges of perceived intensity (21–40; 41–60; 61–80), the ANCOVA showed neither a main effect (*F*(2, 33) = 1.07, *P* = 0.38) nor its interaction with the factor Mind-set (*F*(2, 33) = 0.34, *P* = 0.71), thus suggesting that neither the range of intensity alone nor its combination with the experimental conditions explained the observed effects.

### Auditory intensity and threat

All auditory stimuli were detected by participants during the experiment. The ANCOVA on intensity and threat ratings at S2 revealed that the covariate *pre* was significant for the analysis of intensity and threat (*F*(1, 37) = 224.22, *P*<0.001; pη^2^ = 0.86 and *F*(1, 37) = 676.22, *P*<0.001; pη^2^ = 0.95, respectively). When adjusting for the effect of the ratings obtained in the *pre* blocks, no significant main effect of Mind-set was observed in intensity ratings (*F*(1, 37) = 0.93, *P* = 0.34), but a significant effect was observed in judgments of threat (*F*(1, 37) = 6.21, *P* = 0.02; pη^2^ = 0.14). This effect was accounted for by higher ratings of threat in *MS* trials than *ES* trials, as confirmed by the paired t-tests performed on *post-pre* change scores, in which differences associated with the judgment of threat reached significance (*t*(19) = 4.37, *P*<0.001); the higher relative increase of intensity following *MS* was not significant (*t*(19) = 1.47, *P* = 0.15) (Fig. 2, right panel).

### Nociceptive evoked potentials

**Figure 3:**
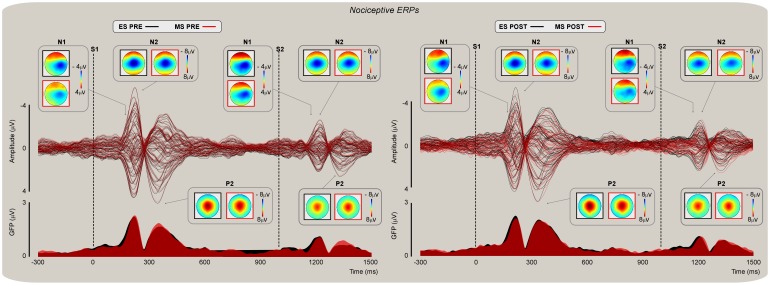
Nociceptive evoked potentials (NEPs). Group-level average scalp topographies of NEPs (upper and lower panel) and global field power (GFP; lower panel) elicited by stimulation of the left hand dorsum before and after mind-set induction (left and right panel respectively). Butterfly plots show ERPs from 60 channels superimposed in 20 participants. NEPs were elicited by pairs of nociceptive stimuli delivered at a fixed 1 s ISI. Representative scalp topographies of each NEP during ES (black) and MS (red) conditions are shown in the insets. Note the amplitude reduction between S1- and S2-related activity.

Grand average waveforms and global field power (GFP) of nociceptive evoked potentials (NEPs) are displayed in Fig. 3. Nociceptive stimuli delivered before (Fig. 3; left panel) and after (Fig. 3; right panel) mind-set induction elicited maximal N2 and P2 waves at the scalp vertex (electrode Cz) and N1 activity corresponding to a lower amplitude topography contralateral to the stimulated body limb.

At Cz, the t-tests performed on S2-ERPs revealed no difference between *MS* and *ES* mind-sets on *pre* and *post* respectively (*t_19_* = −1.40; *P = *0.45; *t_19_* = −2.37; *P = *0.25). However, the within mind-set t-test revealed a significant difference between *pre* and *post* mind-set induction activity during both *MS* (*t_19_* = 3.35; *P* = 0.005) and *ES* (*t_19_* = 3.77; *P* = 0.009) sessions, which was accounted for by lower amplitudes in the N2 wave range after both *MS* and *ES* induction with respect to the *pre* mind-set induction. Importantly though, the ANCOVA on N2 and P2 peak amplitudes confirmed that once *pre* was regressed from *post* no effect of mind-set could be detected (N2: *F*(1, 37) = 0.20, *P* = 0.66; P2: *F*(1, 37) = 0.62, *P* = 0.15, respectively). In both cases, there was an effect of the covariate *pre* (*F*(1, 37) = 46.99, *P*<0.001; pη^2^ = 0.56, and *F*(1, 37) = 26.76, *P*<0.001; pη^2^ = 0.42), suggesting that the N2 wave amplitude reduction observed in *post* was likely due to a general effect of habituation.

At C4, the t-tests performed on S2-ERPs revealed no difference between *MS* and *ES* mind-sets in the *pre* and *post* conditions respectively (*t_19_* = 2.20; *P = *0.28; *t_19_* = −2.06; *P = *0.40). Similarly, at T8 the t-tests performed on S2-ERPs revealed no difference between *MS* and *ES* mind-sets in the *pre* and *post* condition respectively (*t_19_* = −1.85; *P = *0.40; *t_19_* = −0.64; *P = *0.33). Furthermore, the within mind-set t-test also revealed no significant difference between *pre* and *post* at C4 (*ES*: *t_19_* = 1.40; *P = *0.36; *MS*: *t_19_* = −0.32; *P = *0.46) or T8 (*ES*: *t_19_* = −0.004; *P = *0.60; *MS*: *t_19_* = −1.19; *P = *0.50). ANCOVA confirmed no effect in the early N1 peak amplitudes, at either C4 (*F*(1, 37) = 0.26, *P* = 0.61) or T8 *F*(1, 37) = 0.09, *P* = 0.52. The covariate *pre* was significant at both C4 (*F*(1, 37) = 44.65, *P*<0.001; pη^2^ = 0.55) and T8 (*F*(1, 37) = 64.90, *P*<0.001; pη^2^ = 0.64).

Analysis of the N2 amplitude differences as a function of the amplitude response profile (ARP) revealed that there was no significant interaction between ARP and type of Mind-set (*F*(1, 35) = 2.31, *P* = 0.14); however, there was a significant main effect of the ARP (*F*(1, 35) = 8.08, *P*<0.01), which was explained by lower amplitudes in *highs* than *lows*. This finding likely explains why differences in N2 amplitudes were found only during within mind-set t-tests and not between mind-sets t-tests and ANCOVA. This result also suggests the differences found in the N2 amplitudes were not specific to the influence of *MS* or *ES* mind-sets but partly driven by between-subject differences in inherent neural habituation/dishabituation profiles.

### Auditory evoked potentials

**Figure 4:**
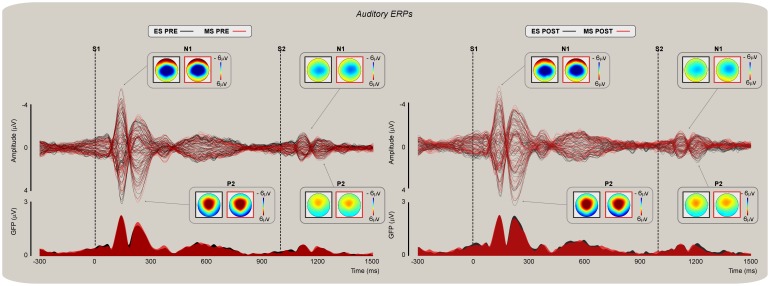
Auditory evoked potentials (AEPs). Group-level averages, scalp topographies, and global field power (GFP) of AEPs elicited by stimulation of the left hand dorsum before and after mindset induction (upper and lower panel respectively). Butterfly plots show ERPs from 60 channels superimposed in 20 participants. ERPs were elicited by pairs of nociceptive stimuli delivered at a fixed 1 s ISI. Representative scalp topographies of each AEP component during ES (black) and MS (red) conditions are shown in the insets. Note the significant amplitude reduction between S1- and S2-related activity.

Grand average waveforms and global field power (GFP) of auditory evoked potentials (AEPs) are displayed in Fig. 4. Auditory stimuli delivered before (Fig. 4; left panel) and after (Fig. 4; right panel) mind-set induction elicited N1 and P2 waves that were maximal at the scalp vertex (electrode Cz). At Cz, the t-tests performed on S2-ERPs revealed no difference between *MS* and *ES* mind-sets in *pre* and *post* respectively (*t_19_* = −2.46; *P = *0.23; *t_19_* = −3.02; *P = *0.17). In addition, no significant difference between *pre* and *post* was revealed by the within mind-set t-test during the *MS* (*t_19_* = 3.21; *P = *0.11) or *ES* (*t_19_* = 3.92; *P = *0.10) sessions. However, similar to the results obtained with the nociceptive ERPs, the ANCOVA on N1 and P2 peak amplitudes confirmed no significant effect of Mind-set (*F*(1, 37) = 2.13, *P* = 0.15 and *F*(1, 37) = 2.17, *P* = 0.15, respectively). In both cases, there was an effect of the covariate *pre* (*F*(1, 37) = 86.50, *P*<0.001; pη^2^ = 0.70, and *F*(1, 37) = 68.02, *P*<0.001; pη^2^ = 0.80).

Analysis of the N1 amplitude differences as a function of ARP revealed no significant interaction between behaving as *lows* or *highs* and type of Mind-set induction (*F*(1, 35) = 1.00, *P* = 0.32) and no significant main effect of the ARP (*F*(1, 35) = 0.71, *P* = 0.40). This control analysis showed that the lack of influence of Mind-set induction on the auditory N1 was not related to individual variability in response amplitude.

### Nociceptive oscillatory activity

**Figure 5:**
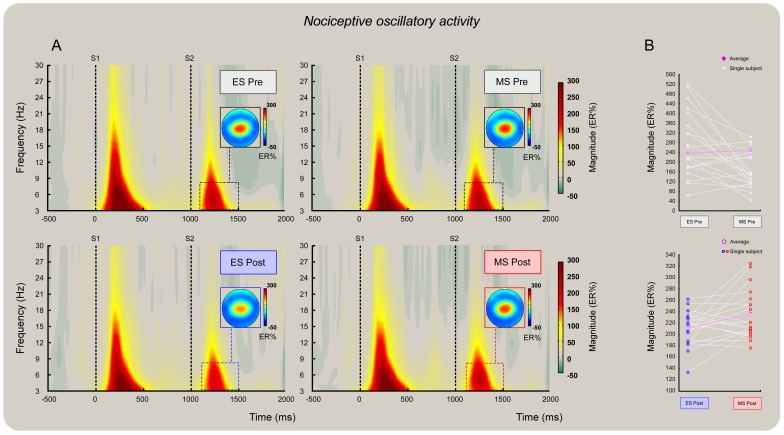
Effect of the two mind-sets induction on the nociceptive S2-ER% oscillatory activity at Cz. Grand average time-frequency representation of nociceptive-related oscillatory activity (as measured at Cz) both before (panel A, top) and after (panel A, bottom) mind-set induction. The a priori identified theta time-frequency ROI was used to extract the “top 10%” of the signal amplitude increase (ER%) relative to the pre-stimulus interval (−0.6 to −0.2 sec before onset of S1). Note the decrease of signal magnitude at S2 following ES mind-set induction (panel A, bottom left). No similar decrease occurred after MS mind-set induction (panel A, bottom right). Panel B: the y axes show single subject and group means of oscillatory amplitude (ER%) before (top) and after (bottom) mind-set induction. Higher ER% magnitude after MS than ES mind-set induction (233±9 vs. 208±6 ER%) (bottom) was detected both by t-test and ANCOVA. ANCOVA revealed that this difference was entirely explained by the modulatory effect of MS on S2 even when regressing out Pre activity. The difference peaked at 262 ms (range: 239–290 ms) and 5 Hz (3.3–6.8 Hz).

**Figure 6:**
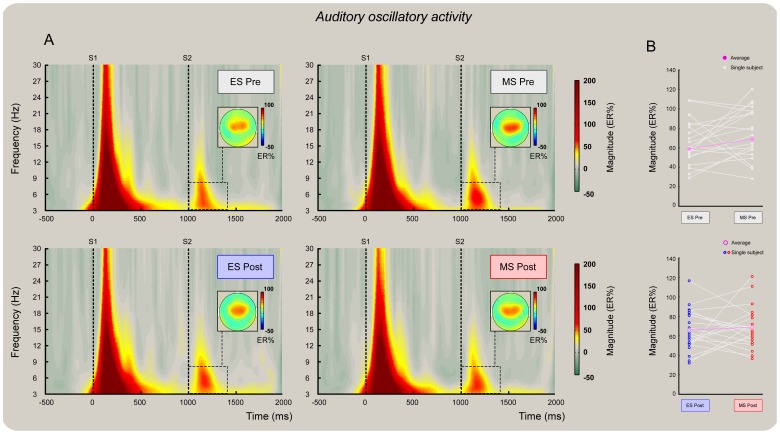
There was no effect of mind-set induction on the auditory S2-ER% oscillatory activity at Cz. Grand average time-frequency representation of nociceptive-related oscillatory activity (as measured at Cz) both before (panel A, top) and after (panel A, bottom) mind-set induction. The a priori identified theta time-frequency ROI was used to extract the “top 10%” of signal amplitude increase (ER%) relative to the pre-stimulus interval (−0.6 to −0.2 sec before the onset of S1). Note the decrease of signal magnitude at S2 following mind-set inductions (panel A, bottom left and right). Panel B: the y axes show single subject and group means of oscillatory amplitude (ER%) before (top) and after (bottom) mind-set inductions.

Grand average spectrograms of nociceptive-related brain activity (as measured at Cz referenced to the common average) both before (Fig. 5, panel A, top) and after (Fig. 5, panel A, bottom) mind-set induction. At Cz, the t-tests performed on the S2-ER% revealed no difference in *pre* mind-set activity (*t_19_* = 0.63; *P = *0.53; Fig. 5, top right graph) but highlighted a significant difference in the *post* mind-set activity at the level of the theta band ROI (*t_19_* = 2.55; *P = *0.02), which was accounted for by a higher ER% magnitude after *MS* than *ES* induction (233±9 vs. 208±6) (Fig. 5, panel B, bottom). This difference peaked at 262 ms (range: 239–290 ms) and at 5 Hz (3.3–6.8 Hz). The within mind-set t-test revealed no significant difference between *pre* and *post* during the *MS* condition (*t_19_* = −0.96; *P = *0.35; Fig. 5, panel A, right), but there was a trend to a significant reduction of S2 ER% magnitude (237±10 vs. 208±6) following *ES* induction (*t_19_* = −2.11; *P = *0.05; Fig. 6, panel A, left). The ANCOVA on ER% S2 magnitude revealed a significant effect of mind-set (*F*(1, 37) = 4.70, *P* = 0.03; pη^2^ = 0.11). Moreover, regressing out the *pre* mind-set activity had no significant effect on the model, i.e., it did not help address the *post* mind-set differences (*F*(1, 37) = 0.33, *P* = 0.57). In other words, the ANCOVA confirmed the difference evidenced by the t-tests, which was entirely explained by higher ER% magnitude in the theta band following the *MS* than the *ES* mind-set (ER% least squares means, *MS* vs. *ES*, 234±8 vs. 207±8; Fig. 5, panel B, bottom).

Analysis of ER% magnitude differences as a function of ARP revealed no significant interaction between behaving as *lows* or *highs* and type of Mind-set (*F*(1, 37) = 0.37, *P* = 0.55) nor a significant main effect of the ARP (*F*(1, 37) = 0.60, *P* = 0.44). At the same time, the introduction of this factor in the ANCOVA model did not affect the significance of the factor Mind-set (*F*(1, 37) = 4.72, *P* = 0.03; pη^2^ = 0.12). This finding suggests that the genuine modulation of oscillatory ER% magnitude was due to the induction of a cognitive mind-set and that the specific effect of mortality salience was reliable regardless of the inherent neural habituation/dishabituation profile of each individual.

### Auditory oscillatory activity

Grand average spectrograms of auditory-related brain activity (as measured at Cz referenced to the common average) both before (Fig. 6; panel A, top) and after (Fig. 6; panel A, bottom) mind-set induction. At Cz, the t-tests performed on S2-ER% revealed no difference in either *pre* mind-set (*t_19_* = 1.55; *P = *0.14) or *post* mind-set activity (*t_19_* = 0.46; *P = *0.65) at the level of the theta band ROI (Fig. 6; panel A, top and bottom, respectively). The within mind-set t-test also revealed no significant difference in either the *ES* (*t_19_* = −0.10; *P = *0.92) or *MS* condition (*t_19_* = 0.73; *P = *0.47) (Fig. 6, panel A, left and right, respectively). The ANCOVA on ER% S2 magnitude confirmed the lack of effect of Mind-set (*F*(1, 37) = 0.38, *P* = 0.54) and no significant contribution of the covariate *pre* to the model variability (*F*(1, 37) = 1.05, *P* = 0.31).

Analysis of ER% magnitude differences as a function of the ARP revealed no significant interaction between behaving as *lows* or *highs* and type of Mind-set induction (*F*(1, 35) = 0.72, *P* = 0.40) or a significant main effect of the ARP (*F*(1, 35) = 1.55, *P* = 0.22). This control analysis showed that the lack of influence of mind-set induction on the auditory theta ER% was not related to individual variability in response profile.

### Covariance of oscillatory activity with subjective ratings and demographics

**Figure 7:**
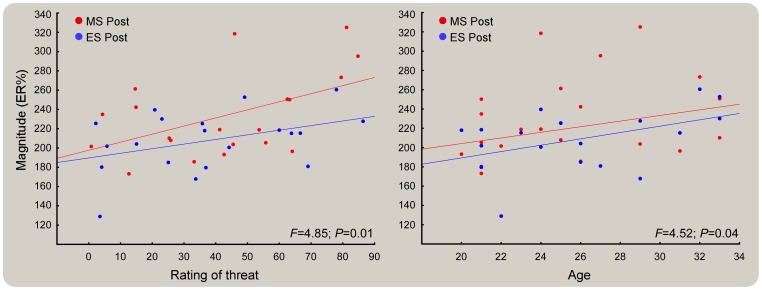
Covariation of nociceptive ER% with subjective ratings (left) and demographics (right). In each scatterplot both MS (red) and ES (blue) conditions are represented with their respective fits. The scatterplot at the left shows that the higher the rating of threat attributed to nociceptive S2, the higher the magnitude of theta activity. More importantly, the different slopes indicate that the increase in the ER% was higher after MS than ES mind-set induction. Similarly, the scatterplot on the right shows that the higher the participant's age the higher the magnitude of theta activity. Note in this case the nearly parallel relationship between the two slopes, which indicates that the effect of age modulated the two mindsets equally.

ANCOVA revealed that, following the induction of mortality salience, the ER% magnitude increased concomitantly with the increase in ratings of threat. That is, the higher the rating of threat attributed to the S2 nociceptive stimulus, the higher the theta ER% magnitude (Fig. 7, left). Moreover, the ER% magnitude increase co-varied with the participants' age, that is, the older the participant, the greater the increase in magnitude regardless of the type of mind-set applied (Fig. 7, right). In addition there was no co-variation of the nociceptive related ER% magnitude with state mood and anxiety measures (lowest *F* = 1.55; lowest *P* = 0.22).

## Discussion

Here, we provide evidence that reminders of one's own mortality have a preferential effect on perceptual judgments (Fig. 2) as well as on cortical spectral activity (Fig. 5) associated with the processing of somatosensory nociceptive input. Furthermore, the effect observed on cortical activity covaried significantly with participants' ratings of threat and their age (Fig. 7). More specifically, we found that the effect of Mind-set on intensity ratings was significant for nociceptive stimuli (Fig. 2, top left) but not for auditory stimuli (Fig. 2, top right). Conversely, the increase in threat ratings following reminders of mortality affected individual's judgments of both nociceptive and auditory stimuli (Fig. 2, bottom left and right). The analyses of ERPs revealed a reduction of the negativity following mind-set induction. However, such decrement became insignificant when the S2 amplitudes recorded before the mind-set induction were regressed out. Furthermore, the effect of Mind-set observed on the nociceptive evoked N2 became insignificant when the variability associated with individual differences in response amplitude was regressed out, thus suggesting that the amplitude reduction was due to habituation and inter-individual variations in response amplitude.

Importantly, the analyses of oscillatory ER% magnitude provided a statistical difference between the two mind-sets. We found higher nociceptive-related theta activity elicited by S2 following reminders of death than following reminders of a failed exam (Fig. 5, panels A and B); no such effect was found in the auditory theta activity (Fig. 6, panels A and B). Note that regressing out the S2 ER% observed before the mind-set induction did not explain the post-induction difference, which was thus entirely explained by higher ER% magnitude in the theta band following mortality salience but not exam salience (Fig. 5, panel B, bottom). Interestingly, this top-down impairment of the expected magnitude suppression at S2 covaried with the participants' perception of threat, that is, the higher the rating of threat attributed to the nociceptive stimulus, the higher the theta ER% magnitude (Fig. 7, left). Moreover, the ER% magnitude increase covaried with the participants' age, that is, the older the participant, the greater the increase in magnitude regardless of the type of mind-set applied (Fig. 7, right).

### The interaction between mortality salience and brain representation of threatening somatosensory input

Here we explored the selective effect of inducing a mortality salience mind-set on the perception of nociceptive stimuli and on the magnitude of evoked potentials and oscillatory EEG activity associated with them. We focused on evoked vertex potentials (N1, N2, and P2 NEPs; N1 and P2 AEPs) and oscillatory responses in the theta band (3–8 Hz). The paired stimulation design served to test whether a significant top-down modulation (indexed by S2-related responses) associated with the contextual relevance of accessibility to death thoughts could revert the amplitude suppression phenomenon associated with the reduced salience of the repeated sensory input.

The effects reported here can be distinguished as mind-set specific (i.e., the modulation of nociceptive theta ER%) vs. mind-set unspecific (i.e., the modulation of NEP N2 and AEP N1). The phase-locked N1, N2, and P2 nociceptive-evoked potentials (especially the vertex N2-P2 waves) include low frequencies in the delta/theta band (1–8 Hz) [15], [47], and particularly in the delta range [47]. Nevertheless, recent studies reported that theta (e.g., [46]) and even alpha spectral activity [45], [55] may contribute to the above components. Thus, there are at least three reasons why the TF representation in the theta band may not entirely account for variations in the magnitude of LEPs. First, different parameters of TF signal decomposition may bring about subtle different results in terms of involved frequencies. Second, and independent of the first reason, the latency and magnitude of the TF decomposition cannot be related only to the contribution of each one of these responses in isolation nor captures solely the phase-locked information (although most of the information represented in the theta band is phase-locked). Third, all sensory evoked responses are contributed by activities in the range of delta and alpha frequencies and not only by the theta frequency band [56]. As we detailed above, the theta oscillatory activity reported in the present study can be considered as a general representation of the late vertex activity evoked in the time domain which only partially represents each specific time-locked nociceptive evoked potential. It has been speculated that activity in the theta range may serve as a biomarker of pain processing [55], [57] and more generally as an index of abnormal neural processing in psychiatric and neurological diseases [58].

It is possible that, as theta activity can be particularly sensitive to the intra- and inter-individual short-term variations of salient sensory information perceived as threatening, and ultimately ensuing in the experience of pain (e.g., [46]), it could be sensitive to the unaware involuntary top-down cognitive/emotional modulation exerted by reminders of mortality. However, according to the TMT theory, such modulation would not be mediated by self-reported negative mood or anxiety. Yet, as correctly argued by Tritt et al. in a recent insightful review [59], this notion cannot rely on a statistical null effect (namely, the absence of differences in ratings of mood between mind-sets). In addition, the idea that defense mechanisms triggered by mortality salience are based on a specific death–related "potential for anxiety" mechanism rather than on a more general anxiety mechanism did not receive enough evidence yet. Therefore, a more biologically plausible explanation should be advocated that is grounded on brain mechanisms evolutionarily developed to resolve uncertainty in the environment (and so avoid unexpected events) [60], basically mediated by a general anxiety system [59].

Despite such theoretical debate, our current results provide evidence in favor of the TMT by showing that the effect of mortality salience on nociceptive theta ER% was not associated with positive and negative affect or anxiety because the self-reported measures did not explain the variance associated with the mind-set effect and did not covary with the observed effects (cf. Results section). Nevertheless, the fact that following mortality salience the ER% magnitude increased concomitantly with the increase in ratings of threat (Fig. 7, left) suggests that the motivational state associated to the reminders of mortality increased arousal and vigilance in the participants. Tritt et al. [59] proposed that biological underpinnings of mortality salience might not be unique but rather a particular expression of a more general set of biological responses to uncertainty. In this respect, the findings obtained in our preliminary survey (see Material S1) support the notion that self-reported measures of general anxiety and emotions may be strongly influenced by mind-sets different from reminders of mortality (e.g. the possibility of becoming paralyzed or being abandoned). These authors suggest that a brain-based anxiety system exists, which is responsible for the biological processes subtending mortality salience and, more generally, threat defense phenomena. This would also explain the specific sensitivity of brain responses to somatosensory threatening stimuli to the top-down modulation exerted by mortality salience. The fact that no mediation of self-reported anxiety on the neural measures was found in the present study, by no means implies that future studies will be unable to identify automatic, implicit and unconscious anxiety mediation of mortality salience effects on the neural correlates of bodily threat.

### Methodological and theoretical considerations

Several aspects of the methods applied here are worth discussion to foster future research in this area. We implemented a calibration procedure in which the intensity of auditory stimulation was adjusted to match the intensity of the laser stimulation. The self-adjusting calibration approach is reported in detail in previous publications [19], [25]. In short, participants were asked to abstain from a separate assessment of auditory sensation and, instead, attempt to equalize their perception of the auditory stimulus in relation to the nociceptive one. However, it must be noted that, despite the attempt to match the perceptual magnitude between the two modalities, the auditory stimulation could not be fully comparable with somatosensory heat stimulation (for it did likely not activate mechano-nociceptors in the ear). In spite of the fact that auditory stimuli were salient and perceived as threatening, they did not induce pain. Thus, whether the effects observed in this study would be replicated using a nociceptive and/or painful stimulus in a sensory modality other than somatosensory remains an open question. However, it should be noted that it may not be possible to induce pain by selectively activating nociceptors in non-somatosensory modalities (i.e. auditory, visual, and olfactory). Despite this criticality, the finding that participants attributed some degree of threat to auditory stimuli, and that this was modulated by reminders of mortality, reflects the success of the perceptual calibration procedure (Fig. 2), thus substantiating the methodological sensitivity of the control sensory stimulation used. Nevertheless, our results cannot provide conclusive modality specific effects, and different sensory stimuli acquiring homeostatic significance/behavioural relevance for the body could exert similar effects at the level of brain responses. In addition, the effect size of the significant difference in the theta oscillatory activity was small (pη^2^ = 0.11), thus suggesting that this finding should be considered as a preliminary observation in need of replication.

The specificity and sensitivity of the effects of mortality salience on theta activity concomitant to somatosensory threatening stimuli is supported by the effects associated with the induction of a failed exam mind-set, a control condition which was suggested to be most comparable to mortality salience across several cognitive/affective dimensions (see Material S1). The biological underpinnings of mortality salience may be not mapped on a specific neural system but rather on a set of areas representing the neural reactivity to uncertainty [59]. It should be emphasized that death was not rated as the worst option in several of the measured circumstances. Indeed, self-reported ratings of negativity, alarm, threat, and significance were higher for other mind-sets (e.g. becoming paralyzed or being abandoned) than for reminders of mortality.

Another important aspect of the methodology adopted in the present study was the use of a within subject design, which contrasts with classical social psychology studies [27], [28]. To the best of our knowledge only two neuroscientific studies [10], [11] applied a within- rather than a between-subjects experimental design. Between-subjects designs do not take into account individual differences in responsiveness to the mind-set induction, hence the participation of an individual in repeated tests in each experimental condition increases the statistical power and precision of the study, as well as it reduces the amount of participants required in a study.

Although the age range of the sample recruited in the present study was limited, an interaction between the cognitive mind-set inductions and the age of the participants (namely, the older the participant the larger the effects of the mind-sets) is consistent with the differential effect of mortality salience across different ages [27], [61]. Yet, future studies with a more representative age group will determine whether the age-related differences reported here are actually a result of developmental changes over the life span and whether the effect may be specific to a cognitive mind-set specifically associated to reminders of mortality or whether it would be an unspecific effect, as observed in the current study.

To conclude, our findings support the hypothesis that reminders of mortality have a modulatory effect on the perception of threatening somatosensory stimuli and their associated neural responses. Importantly, this effect becomes stronger the more the stimuli are judged as threatening, suggesting an influence of death-related thoughts on somatosensory representation.

## Supporting Information

Material S1 Preliminary survey results showing self-report mind-set categorization in a sample of 100 respondents.
(DOC)
