# Supplementary material for: Thoughts of Death Modulate Psychophysical and Cortical Responses to Threatening Stimuli
Source: PLoS One. 2014 Nov 11;9(11):e112324. doi: 10.1371/journal.pone.0112324 (PMC4227888; doi:10.1371/journal.pone.0112324)
Supplement: Material S1 — Preliminary survey results showing self-report mind-set categorization in a sample of 100 respondents. (DOC) [file pone.0112324.s001.doc]

**Supplementary material (S1)**

Preliminary survey results showing self-report mind-set categorization in a sample of 100 respondents.

Volunteers were asked to judge the following mind-sets across several cognitive/emotional dimensions:

- The thought of one's own death.
- The thought of failing an important exam.
- The thought of one's own paralysis.
- The thought of being abandoned by relatives and friends.
- The thought of watching television (TV).

The cognitive/emotional dimensions were explored by the following questions:

- How arousing do you find the thought of your death?
- How negative do you find the thought of your death?
- How positive do you find the thought of your death?
- How threatening do you find the thought of your death?
- How frightening do you find the thought of your death?
- How alarming do you find the thought of your death?
- How puzzling do you find the thought of your death?
- How insignificant do you find the thought of your death?
- How relaxing do you find the thought of your death?
- How pleasant do you find the thought of your death?

All questions were asked in randomised order using the utilities provided by SurveyMonkey® (<http://it.surveymonkey.com/>). Volunteers could reply to each question using a Likert scale (Not at all/slightly=1, a little=2, moderately=3, much/highly=4, extremely=5).

Distribution of the variables was skewed and strongly non-normal (Kolmogorov-Smirnov and Shapiro-Wilk tests; p value <0.05). Therefore, in accordance with the Likert-type ordinal nature of the survey, we used a non-parametric approach by combining Friedman’s ANOVA and Wilcoxon’s Matched Pairs Test to analyze differences across mind-sets for each cognitive/emotional dimension.

Mean rank and standard deviation (SD) and Wilcoxon’s Test results are reported in tables (Table 1-10).

Results showed a significant difference in arousal ratings between mind-sets (*χ*^2^(4.396)=85.45; *P*<0.001). This difference was accounted for by lower ratings of arousal for the thought of watching TV than all other mind-sets (Table 1). A significant difference emerged in the valence ratings between mind-sets for ratings of both negative and positive valence (*χ*^2^(4.396)=208.57; *P*<0.001; *χ*^2^(4.396)=160.92; *P*<0.001). This difference was accounted for by lower negative ratings for watching TV and higher ratings for paralysis and being abandoned. Crucially, negative ratings did not differ between thinking of death and failing an exam (Table 2).

On the other hand, although almost none of the mind-sets were considered positive, the clear absence of difference between thinking of death and failing an exam was confirmed (Table 3). A significant difference was found for ratings of threat, fear and alarm (*χ*^2^(4.396)=178.87; *P*<0.001; *χ*^2^(4.396)=236.22; *P*<0.001; *χ*^2^(4.396)=210.76; *P*<0.001). This difference was accounted for by significantly higher ratings of threat for the thought of becoming paralysed and low ratings of threat for watching TV. Threat was not significantly different between thinking of death and thinking of failing an exam and of being abandoned (Table 4). Ratings of fear were significantly different across all comparisons and resembled the profile observed for threat ratings, that is, higher ratings of fear for the thought of becoming paralysed and low ratings of fear for watching TV (Table 5). Ratings of worry were significantly different across all comparisons except for thinking of death and thinking of failing an exam (Table 6). Again, ratings of puzzlement were also significantly different (*χ*^2^(4.396)=205.86; *P*<0.001) across all comparisons except for thinking of death and thinking of failing an exam (Table 7). Ratings of insignificance were significantly different (*χ*^2^(4.396)=124.02; *P*<0.001). This difference was accounted for by higher ratings of insignificance for watching TV with respect to all other mind-sets. Furthermore, failing an exam was considered less meaningful than being abandoned or becoming paralysed (Table 8).

Ratings of relaxation and pleasantness were significantly different across mind-sets (*χ*^2^(4.396)=259.37; *P*<0.001; *χ*^2^(4.396)=276.90; *P*<0.001). The difference was accounted for by higher ratings of relaxation for watching TV than all other mind-sets and lower ratings of relaxation for failing an exam, becoming paralysed and being abandoned with respect to thinking about death (Table 9). Similarly, differences in pleasantness were accounted for by higher ratings for watching TV than all other mind-sets and lower ratings for failing an exam and becoming paralysed with respect to thinking of death (Table 10).

**Tables**

Median and standard deviation (SD) and *P* values (*P*<0.05 = *; *P*<0.01 = **; *P*<0.001 = ***) are provided for each of the ten dimensions investigated.

Table 1. *Ratings of arousal*.

| Death | Exam failure | Paralysis | Abandonment | Watching TV |
| --- | --- | --- | --- | --- |
| 2.77 (1.43) | 2.75 (1.37) | 2.82 (1.65) | 2.93 (1.45) | 1.40 (0.70) |
|  |  |  |  |  |
| Wilcoxon Matched Pairs Test | | | | |
|  |  | *T* | *Z* | *P* |
| Death vs. Exam |  | 763.0 | 0.06 | 0.95 |
| Death vs. Paralysis |  | 610.5 | 0.26 | 0.79 |
| Death vs. Abandonment |  | 598.0 | 1.24 | 0.21 |
| Death vs. Watching TV |  | 172.5 | 6.48 | <0.001*** |
| Exam vs. Paralysis |  | 746.0 | 0.42 | 0.67 |
| Exam vs. Abandonment |  | 544.5 | 1.32 | 0.18 |
| Exam vs. Watching TV |  | 221.5 | 6.42 | <0.001*** |
| Paralysis vs. Abandonment |  | 644.5 | 0.63 | 0.53 |
| Paralysis vs. Watching TV |  | 154.0 | 6.23 | <0.001*** |
| Abandonment vs. Watching TV |  | 163.0 | 6.66 | <0.001*** |

Table 2. *Ratings of negativity*.

| Death | Exam failure | Paralysis | Abandonment | Watching TV |
| --- | --- | --- | --- | --- |
| 3.36 (1.20) | 3.52 (0.97) | 4.40 (0.83) | 4.16 (0.93) | 1.85 (1.12) |
|  |  |  |  |  |
| Wilcoxon Matched Pairs Test | | | | |
|  |  | *T* | *Z* | *P* |
| Death vs. Exam |  | 1039.0 | 1.0 | 0.37 |
| Death vs. Paralysis |  | 265.0 | 6.12 | <0.001*** |
| Death vs. Abandonment |  | 395.0 | 5.15 | <0.001*** |
| Death vs. Watching TV |  | 172.5 | 6.29 | <0.001*** |
| Exam vs. Paralysis |  | 257.0 | 5.93 | <0.001*** |
| Exam vs. Abandonment |  | 550.0 | 4.51 | <0.001*** |
| Exam vs. Watching TV |  | 124.5 | 7.68 | <0.001*** |
| Paralysis vs. Abandonment |  | 642.0 | 2.0 | 0.04* |
| Paralysis vs. Watching TV |  | 50.5 | 8.18 | <0.001*** |
| Abandonment vs. Watching TV |  | 48.5 | 8.14 | <0.001*** |

Table 3. *Ratings of positivity*.

| Death | Exam failure | Paralysis | Abandonment | Watching TV |
| --- | --- | --- | --- | --- |
| 1.26 (0.56) | 1.22 (0.69) | 1.02 (0.14) | 1.09 (0.32) | 2.00 (0.86) |
|  |  |  |  |  |
| Wilcoxon Matched Pairs Test | | | | |
|  |  | *T* | *Z* | *P* |
| Death vs. Exam |  | 142.0 | 0.55 | 0.58 |
| Death vs. Paralysis |  | 0.0 | 3.72 | <0.001*** |
| Death vs. Abandonment |  | 34.0 | 2.65 | 0.008** |
| Death vs. Watching TV |  | 180.0 | 5.67 | <0.001*** |
| Exam vs. Paralysis |  | 6.0 | 2.76 | 0.005** |
| Exam vs. Abandonment |  | 7.0 | 2.31 | 0.02* |
| Exam vs. Watching TV |  | 271.5 | 5.50 | <0.001*** |
| Paralysis vs. Abandonment |  | 4.0 | 1.96 | 0.05 |
| Paralysis vs. Watching TV |  | 0.0 | 7.06 | <0.001*** |
| Abandon vs. Watching TV |  | 0.0 | 6.79 | <0.001*** |

Table 4. *Ratings of threat*.

| Death | Exam failure | Paralysis | Abandonment | Watching TV |
| --- | --- | --- | --- | --- |
| 3.09 (1.46) | 2.81 (1.20) | 4.01 (1.07) | 3.36 (1.34) | 1.32 (0.80) |
|  |  |  |  |  |
| Wilcoxon Matched Pairs Test | | | | |
|  |  | *T* | *Z* | *P* |
| Death vs. Exam |  | 834.0 | 1.56 | 0.11 |
| Death vs. Paralysis |  | 299.0 | 5.34 | <0.001*** |
| Death vs. Abandonment |  | 996.5 | 1.61 | 0.10 |
| Death vs. Watching TV |  | 137.5 | 6.92 | <0.001*** |
| Exam vs. Paralysis |  | 172.0 | 6.61 | <0.001*** |
| Exam vs. Abandonment |  | 664.5 | 3.38 | <0.001*** |
| Exam vs. Watching TV |  | 176.5 | 6.92 | <0.001*** |
| Paralysis vs. Abandonment |  | 393.5 | 4.55 | <0.001*** |
| Paralysis vs. Watching TV |  | 27.5 | 8.36 | <0.001*** |
| Abandon vs. Watching TV |  | 147.0 | 7.48 | <0.001*** |

Table 5. *Ratings of fear*.

| Death | Exam failure | Paralysis | Abandonment | Watching TV |
| --- | --- | --- | --- | --- |
| 3.33 (1.33) | 2.77 (1.20) | 4.42 (0.79) | 3.90 (1.10) | 1.26 (0.70) |
|  |  |  |  |  |
| Wilcoxon Matched Pairs Test | | | | |
|  |  | *T* | *Z* | *P* |
| Death vs. Exam |  | 832.0 | 3.13 | 0.002** |
| Death vs. Paralysis |  | 105.0 | 6.18 | <0.001*** |
| Death vs. Abandonment |  | 735.5 | 3.25 | 0.001** |
| Death vs. Watching TV |  | 48.0 | 7.85 | <0.001*** |
| Exam vs. Paralysis |  | 58.0 | 7.70 | <0.001*** |
| Exam vs. Abandonment |  | 249.0 | 6.36 | <0.001*** |
| Exam vs. Watching TV |  | 172.5 | 7.01 | <0.001*** |
| Paralysis vs. Abandonment |  | 287.0 | 4.40 | <0.001*** |
| Paralysis vs. Watching TV |  | 4.5 | 8.62 | <0.001*** |
| Abandon vs. Watching TV |  | 20.0 | 8.30 | <0.001*** |

Table 6. *Ratings of alarm*.

| Death | Exam failure | Paralysis | Abandonment | Watching TV |
| --- | --- | --- | --- | --- |
| 3.19 (1.31) | 2.96 (1.23) | 4.19 (0.96) | 3.72 (1.19) | 1.34 (0.79) |
|  |  |  |  |  |
| Wilcoxon Matched Pairs Test | | | | |
|  |  | *T* | *Z* | *P* |
| Death vs. Exam |  | 1173.5 | 1.33 | 0.18 |
| Death vs. Paralysis |  | 152.5 | 6.01 | <0.001*** |
| Death vs. Abandonment |  | 725.5 | 3.16 | 0.001** |
| Death vs. Watching TV |  | 126.5 | 7.40 | <0.001*** |
| Exam vs. Paralysis |  | 224.0 | 6.56 | <0.001*** |
| Exam vs. Abandonment |  | 578.0 | 4.47 | <0.001*** |
| Exam vs. Watching TV |  | 135.0 | 7.30 | <0.001*** |
| Paralysis vs. Abandonment |  | 468.0 | 3.29 | 0.001** |
| Paralysis vs. Watching TV |  | 0.0 | 8.55 | <0.001*** |
| Abandon vs. Watching TV |  | 66.5 | 8.02 | <0.001*** |

Table 7. *Ratings of puzzlement*.

| Death | Exam failure | Paralysis | Abandonment | Watching TV |
| --- | --- | --- | --- | --- |
| 3.00 (1.29) | 2.76 (1.19) | 4.10 (0.99) | 3.59 (1.21) | 1.31 (0.78) |
|  |  |  |  |  |
| Wilcoxon Matched Pairs Test | | | | |
|  |  | *T* | *Z* | *P* |
| Death vs. Exam |  | 1201.5 | 1.18 | 0.24 |
| Death vs. Paralysis |  | 217.0 | 6.23 | <0.001*** |
| Death vs. Abandonment |  | 744.5 | 3.59 | <0.001*** |
| Death vs. Watching TV |  | 230.0 | 7.00 | <0.001*** |
| Exam vs. Paralysis |  | 140.0 | 7.16 | <0.001*** |
| Exam vs. Abandonment |  | 317.5 | 5.13 | <0.001*** |
| Exam vs. Watching TV |  | 136.0 | 6.93 | <0.001*** |
| Paralysis vs. Abandonment |  | 549.0 | 3.68 | <0.001*** |
| Paralysis vs. Watching TV |  | 12.0 | 8.42 | <0.001*** |
| Abandon vs. Watching TV |  | 50.0 | 7.94 | <0.001*** |

Table 8. *Ratings of insignificance*.

| Death | Exam failure | Paralysis | Abandonment | Watching TV |
| --- | --- | --- | --- | --- |
| 1.43 (0.91) | 1.56 (0.83) | 1.27 (0.78) | 1.35 (0.82) | 2.81 (1.40) |
|  |  |  |  |  |
| Wilcoxon Matched Pairs Test | | | | |
|  |  | *T* | *Z* | *P* |
| Death vs. Exam |  | 440.0 | 1.10 | 0.27 |
| Death vs. Paralysis |  | 180.0 | 1.33 | 0.18 |
| Death vs. Abandonment |  | 155.5 | 0.80 | 0.42 |
| Death vs. Watching TV |  | 321.5 | 6.23 | <0.001*** |
| Exam vs. Paralysis |  | 271.0 | 2.61 | 0.008** |
| Exam vs. Abandon |  | 303.0 | 2.05 | 0.04* |
| Exam vs. Watching TV |  | 381.5 | 6.10 | <0.001*** |
| Paralysis vs. Abandonment |  | 183.5 | 0.73 | 0.46 |
| Paralysis vs. Watching TV |  | 143.0 | 6.90 | <0.001*** |
| Abandon vs. Watching TV |  | 229.0 | 6.60 | <0.001*** |

Table 9. *Ratings of relaxation*.

| Death | Exam failure | Paralysis | Abandonment | Watching TV |
| --- | --- | --- | --- | --- |
| 1.21 (0.51) | 1.08 (0.37) | 1.02 (0.14) | 1.09 (0.40) | 2.46 (0.98) |
|  |  |  |  |  |
| Wilcoxon Matched Pairs Test | | | | |
|  |  | *T* | *Z* | *P* |
| Death vs. Exam |  | 27.0 | 2.55 | 0.01* |
| Death vs. Paralysis |  | 0.0 | 3.52 | <0.001*** |
| Death vs. Abandonment |  | 48.0 | 2.13 | 0.03* |
| Death vs. Watching TV |  | 94.5 | 7.32 | <0.001*** |
| Exam vs. Paralysis |  | 5.0 | 1.52 | 0.13 |
| Exam vs. Abandonment |  | 17.0 | 0.14 | 0.89 |
| Exam vs. Watching TV |  | 105.0 | 7.44 | <0.001*** |
| Paralysis vs. Abandonment |  | 2.5 | 1.68 | 0.09 |
| Paralysis vs. Watching TV |  | 0.0 | 7.87 | <0.001*** |
| Abandon vs. Watching TV |  | 92.5 | 7.44 | <0.001*** |

Table 10. *Ratings of pleasantness*.

| Death | Exam failure | Paralysis | Abandonment | Watching TV |
| --- | --- | --- | --- | --- |
| 1.14 (0.43) | 1.01 (0.10) | 1.03 (0.30) | 1.06 (0.28) | 2.34 (0.87) |
|  |  |  |  |  |
| Wilcoxon Matched Pairs Test | | | | |
|  |  | *T* | *Z* | *P* |
| Death vs. Exam |  | 5.0 | 2.67 | 0.007** |
| Death vs. Paralysis |  | 12.0 | 2.12 | 0.03* |
| Death vs. Abandonment |  | 16.5 | 1.76 | 0.08 |
| Death vs. Watching TV |  | 48.0 | 7.64 | <0.001*** |
| Exam vs. Paralysis |  | 0.0 | 7.41 | <0.001*** |
| Exam vs. Abandonment |  | 3.0 | 1.57 | 0.11 |
| Exam vs. Watching TV |  | 0.0 | 7.91 | <0.001*** |
| Paralysis vs. Abandonment |  | 6.0 | 0.94 | 0.34 |
| Paralysis vs. Watching TV |  | 21.5 | 7.81 | <0.001*** |
| Abandon vs. Watching TV |  | 42.0 | 7.67 | <0.001*** |
